# Effectiveness of Front-Of-Pack Nutrition Labels in French Adults: Results from the NutriNet-Santé Cohort Study

**DOI:** 10.1371/journal.pone.0140898

**Published:** 2015-10-28

**Authors:** Pauline Ducrot, Caroline Méjean, Chantal Julia, Emmanuelle Kesse-Guyot, Mathilde Touvier, Léopold Fezeu, Serge Hercberg, Sandrine Péneau

**Affiliations:** 1 Université Paris 13, Equipe de Recherche en Epidémiologie Nutritionnelle, Centre de Recherche en Epidémiologie et Statistiques, Inserm (U1153), Inra (U1125), Cnam, COMUE Sorbonne Paris Cité, Bobigny, France; 2 Département de Santé Publique, Hôpital Avicenne, Bobigny Cedex, France; University of Bath, UNITED KINGDOM

## Abstract

**Background:**

To date, no consensus has emerged on the most appropriate front-of-pack (FOP) nutrition label to help consumers in making informed choices. We aimed to compare the effectiveness of the label formats currently in use: nutrient-specific, graded and simple summary systems, in a large sample of adults.

**Methods:**

The FOP label effectiveness was assessed by measuring the label acceptability and understanding among 13,578 participants of the NutriNet-Santé cohort study, representative of the French adult population. Participants were exposed to five conditions, including four FOP labels: Guideline Daily Amounts (GDA), Multiple Traffic Lights (MTL), 5-Color Nutrition Label (5-CNL), Green Tick (Tick), and a “no label” condition. Acceptability was evaluated by several indicators: attractiveness, liking and perceived cognitive workload. Objective understanding was assessed by the percentage of correct answers when ranking three products according to their nutritional quality. Five different product categories were tested: prepared fish dishes, pizzas, dairy products, breakfast cereals, and appetizers. Differences among the label effectiveness were compared with chi-square tests.

**Results:**

The 5-CNL was viewed as the easiest label to identify and as the one requiring the lowest amount of effort and time to understand. GDA was considered as the least easy to identify and to understand, despite being the most attractive and liked label. All FOP labels were found to be effective in ranking products according to their nutritional quality compared with the “no label” situation, although they showed differing levels of effectiveness (p<0.0001). Globally, the 5-CNL performed best, followed by MTL, GDA and Tick labels.

**Conclusions:**

The graded 5-CNL label was considered as easy to identify, simple and rapid to understand; it performed well when comparing the products’ nutritional quality. Therefore, it is likely to present advantages in real shopping situations where choices are usually made quickly.

## Introduction

**Fig 1:**
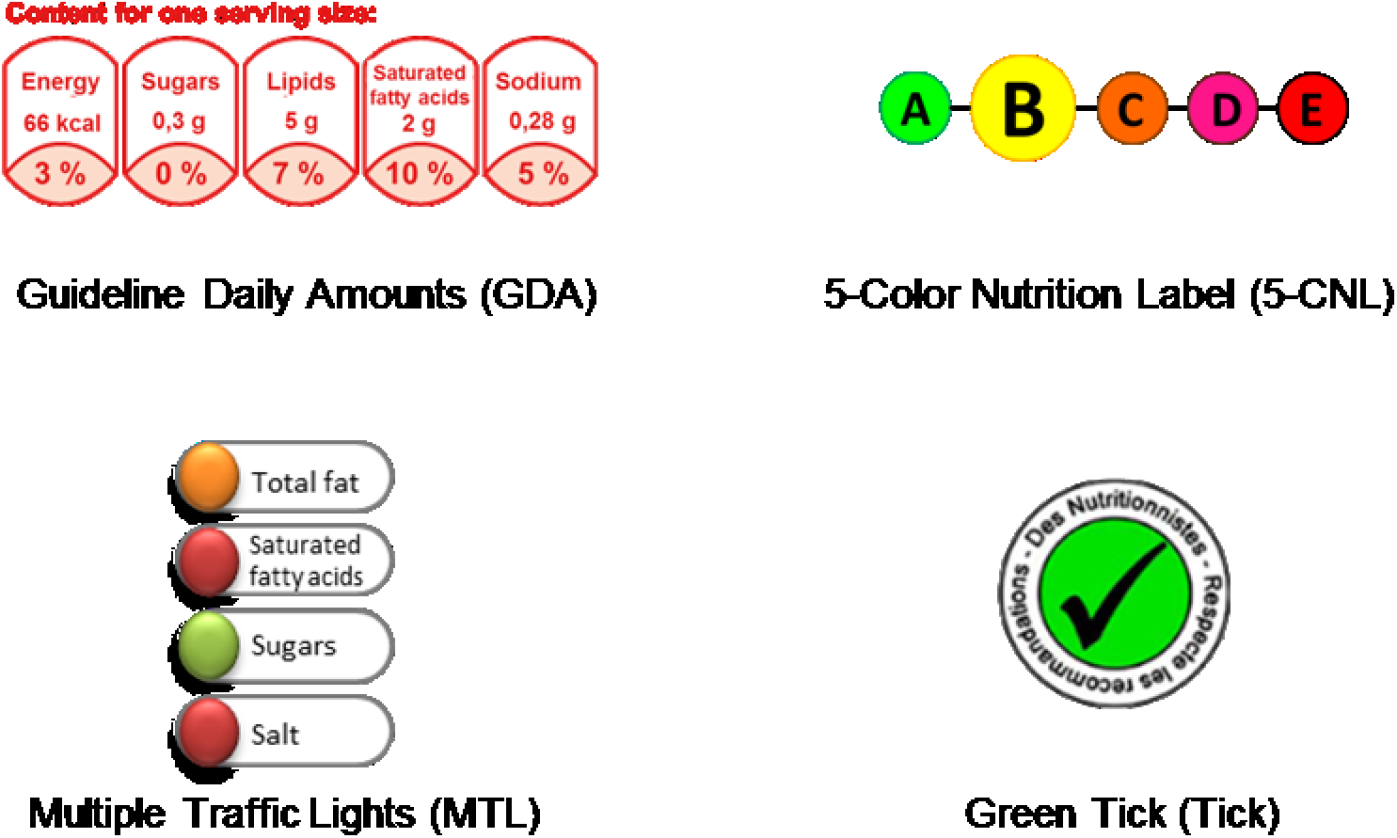
Nutrition labels used in the study.

Helping consumers to make healthier food choices is considered as a key lever of public health policies to improve nutritional status of individuals and prevent chronic diseases [1]. To achieve this, one proposed tool is introducing a simplified nutrition labeling system on the front of each food package, providing simplified information on nutritional content at a glance, along with back-of-pack detailed energy and nutrient content information. This measure is considered useful to enlighten consumers on the nutritional quality of foodstuffs at the time of purchase and has been proven to be effective to identify healthier food products [2–8]. Moreover, this measure is considered as a successful way to promote the improvement of the foods’ nutritional values by the industry [9,10]. Currently, the front-of-pack (FOP) labeling systems can be divided into nutrient-specific and summary labels [3,11–21] (see examples in **Fig 1**). Nutrient-specific labels display nutritional information on several nutrients, such as the Guideline Daily Amounts (GDA) and the Multiple Traffic Lights (MTL) [22,23]. Summary labels provide information about the overall nutritional quality of the product and are generally based on nutrient profiling systems. Summary labels are divided into simple formats such as the Keyhole symbol displayed on “healthier” products and graded formats such as Guiding stars, which display a ranking of zero to three stars [24,25]. To date, no single format has emerged as the most effective in guiding consumers to healthier food choices [5,14]. Some studies have shown that the MTL label was effective in the general population when comparing products in terms of their healthfulness [4,11,12]. In turn, other investigations have suggested that in specific population subgroups, such as those with low formal education, simpler FOP formats performed better than more complex systems [5,6,14,18]. Previous studies have concluded that in order to be effective for everyone, the system has to be simple, clearly visible, recognizable and rapidly comprehensible [5,14,18].

In a recent review of the literature, the Institute of Medicine’s Committee on Examination of FOP Nutrition Rating Systems and Symbols recommended the adoption of graded summary labels that are simple to understand and offer nutritional guidance by using a scaled approach [14]. Following these recommendations, a new graded summary system was developed in France and proposed for use on commercial food items: the 5-Color Nutrition Label (5-CNL) [26]. Relatively few studies, however, have assessed consumer understanding of nutritional quality of foodstuffs based on graded summary systems [5], which highlights the need to evaluate their effectiveness.

In their conceptual framework, Grunert and Wills suggested that the use of FOP labels depended on consumer search, exposure and perception of the displayed information [4]. Further, perception leads to processing of the information, which can be influenced by the acceptability and understanding of the label [4,17]. Only a few studies have evaluated both acceptability and understanding, although both are likely to considerably affect label use. According to a framework proposed by Nielsen [27], label acceptability includes consumer liking, attractiveness and perceived cognitive workload. Liking for a label gathers different notions such as subjective preference for a label [4], but also whether an individual would actually like to have this label on the food packages. Attractiveness for a label is influenced by the ease of identification (i.e. how easily individuals can perceive the label on the package) or the perceived reliability of the label, which could, for example, be enhanced by institutional or scientific endorsement [28]. Perceived cognitive workload refers to the perception of potential format defects leading to difficulty in understanding or discomfort [17]. To date, only one previous study evaluated label acceptability using such an overall approach (i.e. investigating liking, attractiveness and perceived cognitive workload). This study did not identify a clear superiority of one particular format of FOP label [17]. However, this latter study did not include all label formats currently in use, such as the GDA and a graded summary format. Given the fact that summary labels provide synthetized information, it can be hypothesized that such summary labels are perceived as easier to understand in terms of cognitive perceived workload. Nonetheless, liking and attractiveness appear to be more subjective perceptions and are therefore more likely to vary across individuals.

Understanding can be assessed using either subjective or objective measures. Subjective understanding corresponds to the extent to which consumers believe they have understood a label. Objective understanding is whether the information understood by consumers is compatible with the information provided by the label [4] and has been proven to enable a more accurate measurement since self-reported understanding is susceptible to overestimations [4]. Evidence in the literature regarding the understanding of FOP labels by consumers is not clear-cut [2,4,5].

The present study aimed to evaluate the effectiveness of the different formats of FOP labels currently in use worldwide (i.e. nutrient-specific, graded, and simple summary labels), by evaluating both participant acceptability (including liking, attractiveness and perceived cognitive workload) and objective understanding. The potential relationship between socio-demographic characteristics and understanding was also investigated.

## Methods

### Study population

The NutriNet-Santé study (https://info.etude-nutrinet-sante.fr) is an ongoing web-based prospective observational cohort study launched in France in May 2009 with a scheduled follow-up of 10 years. It aims to investigate the relationship between nutrition and chronic disease risk, as well as the determinants of dietary behavior and nutritional status. The study was implemented in the French general population (internet-using adult volunteers, aged ≥18years). The rationale, design and methodology of the study have been fully described elsewhere [29]. In brief, to be considered as included into the study, participants have to complete a baseline set of self-administered, web-based questionnaires assessing dietary intake, physical activity, anthropometric characteristics, lifestyle, socioeconomic conditions and health status. As part of the follow-up, participants are asked to complete the same set of questionnaires each year. Moreover, each month, participants are invited by e-mail to fill in optional questionnaires related to dietary intake, determinants of eating behaviors, nutritional and health status. This study is conducted in accordance with the Declaration of Helsinki, and all procedures were approved by the Institutional Review Board of the French Institute for Health and Medical Research (IRB Inserm n°0000388FWA00005831) and the *Commission Nationale de l’Informatique et des Libertés* (CNIL n°908450 and n°909216). All participants provided informed consent with an electronic signature. This study is registered in EudraCT (n°2013-000929-31).

### Label formats

Four labels (**Fig 1**) were tested in the study, providing simple or more detailed nutritional information along with a simple positive, neutral, or negative opinion of the product. In the introduction to the questionnaire, the different labels were presented and briefly explained to the participants. A detailed description of the labels used in the present study is provided below.

Nutrient-specific formats:

1. Guideline Daily Amounts (GDA): this label indicates the kilocalories and the amount of fat, saturated fatty acids, sugars and sodium in gram per portion as well as their corresponding contribution (in percentages) to the guideline-based daily intakes [22]. The guideline-based daily intakes indicate the recommended intake in kilocalories (kcal/day) and nutrients (g/day). The GDA information was calculated by using the Food and Drink Federation’s guiding principles and was based on the average nutrient requirements for an adult woman. This label can be found on most of the food packaging on the French market, following the voluntary initiative of manufacturers.
2. Multiple Traffic Lights (MTL): this label introduced by the UK Food Standard Agency (FSA), provides an evaluation of nutrient content regarding fat, saturated fatty acids, sugars and sodium. Depending on the quantity of the nutrient in the product (high, medium, low), a color is attributed to each nutrient (red, amber, green, respectively). The colors reflect the concentration in grams per 100 g or per 100 ml of product and the criteria of the FSA were applied to assign the color codes [23].

Summary formats:

3 Five-Color Nutrition Label (5-CNL): this label was proposed to be introduced to the French market to guide consumer food choices [26]. Based on the FSA nutrient profiling system [30], used by the Office of Communication (OfCom) for regulation of advertising to children in the UK, and with specific adaptations for cheese and added fat, this label provides information about the overall nutritional quality of a given food item. The label is represented by a scale of five colors (from green to red) with corresponding letters (from A to E) [26]. The colors were attributed depending on the score of each food given by the FSA nutrient profiling system (**S1 Fig**) (26): ‘Green’ (-15 to -2 points), ‘Yellow’ (-1 to 3 points), ‘Orange’ (4 to 11 points), ‘Pink’ (12 to 16 points) and ‘Red’ (> = 17 points) [26].
4 Green Tick (Tick): this label was derived from the “Keyhole” and “Pick the Tick” symbols, developed by the Swedish Food Administration and the Heart Foundation in Australia and New Zealand, respectively [25,31]. It reflects the overall nutritional quality of the food item and appears only on the healthier products within a food family. The Tick label was attributed to products assigned to the green or yellow categories of the 5-CNL.

Reference

5 No label: a situation without any FOP labels was used as reference.

### Data collection

Objective understanding and acceptability of the different labels were assessed in July 2014 via a web-based questionnaire.

#### Acceptability of front-of-pack labels

Label acceptability was evaluated via several indicators: liking, attractiveness and perceived cognitive workload. These dimensions were inspired by the framework of system acceptability developed by Nielsen [27], which has already been applied to FOP labels by Méjean et al. [17–19]. For this test, participants were asked to select the label that best corresponded to the proposed statements (one possible answer only). An option “none of these labels” was also proposed.

Liking was assessed by asking participants to choose i. Their preferred label (‘This is my preferred label’), ii. The one they least appreciated (‘This is the label I appreciate the least’), iii. The one they wanted to see on the front of packages (‘I want to see this label on the front of packages’) and iv. The one they found the most useful in choosing healthy products (‘This FOP label is the best to help me to choose healthy products’).

Attractiveness was assessed by the perception of the quality of each label and in particular by i. Its perceived contribution to needed information (‘This FOP label provides me with the information I need‘), ii. Ease of identification (‘This FOP label is easy to identify’) and iii. Reliability (‘This FOP label provides reliable information’ and ‘I can rely on this label’).

Perceived cognitive workload was assessed by three indicators: i. Complexity of understanding (‘This FOP label is too complex to understand’ and ‘This FOP label is easy to understand’), ii. Perceived time needed for interpreting the label (‘This FOP label takes too long to understand’ and ‘This FOP label permits rapid understanding of the information’) and iii. Discomfort occasioned by the label (‘This FOP label makes me uncomfortable’).

#### Objective understanding of front-of-pack labels

Objective understanding was examined in five different conditions: four alternatives corresponding to the four different FOP labels and one alternative without any labels. Subjects were asked to rank three products according to their nutritional quality. For this purpose, participants were shown pictures of three products of the same food category, with the corresponding FOP label in the front of the package and were asked: “From your point of view, rank these products according to their nutritional quality (from the lowest nutritional quality to the highest).” The “I don’t know” option was also included. The key criterion for the selection of these three food products was that they had to have distinct (i.e. sufficiently different) nutritional qualities, thus making it possible to rank them by using the labels (except for the Tick label which was not designed to allow graded ranking). No other information on nutrition facts was provided and any quality labels (e.g. organic certification) were removed from the images of the products. Ranking was considered correct if the three products were ranked in the expected order (i.e. according to information on nutritional quality provided by the labels), without any “I don’t know” responses. The products were chosen by nutrition experts so that ranking provided by the labels was consistent with nutrition guidelines and that all tested FOP label led to the same expected ranking (**S1 Appendix**). For the Tick which is only stamped on healthier products, the expected ranking was that provided by other labels.

Five different product categories were tested: prepared fish dishes, pizzas, dairy products, breakfast cereals and appetizers. Each participant was exposed to the five FOP label conditions and to the five product categories. To avoid any potential effect of the product category on the understanding of the FOP label (i.e. due to better knowledge of specific products), each label was associated with all product categories. Each participant was shown five label/product combinations where all five FOP label conditions and five product categories were represented. A rotation system based on a Latin Square design was employed to ensure that an equal number of participants were shown each label/product category combination while controlling for potential order effect of the labels. Thus, a total of 25 different versions of the questionnaire were used. For example, one participant was shown the 5-CNL on frozen prepared fish dishes, and MTL on fresh pizzas, while another participant was shown the 5-CNL on fresh pizzas and MTL on dairy products, etc. In addition, one respondent would be shown the 5-CNL first, while another participant would be shown the MTL first, etc.

#### Sociodemographic data

At baseline and annually thereafter, participants in the NutriNet-Santé Study, are asked to provide socio-demographic data including sex, age (18–30, 30–50, 50–65, >65), educational level (up to secondary, some college or university degree) and occupational category (blue-collar, manual workers; intermediate profession/office work; self-employed, farmer; managerial staff; without any professional activity (student, unemployed); retired). For each participant, the most up-to-date available socio-demographic data were used.

### Statistical analyses

We performed analyses using data from participants of the NutriNet-Santé cohort who had completed the questionnaire on FOP labels. We excluded participants who had responded “I don’t know” to more than two thirds of the cases. We compared included and excluded participants using Student’s t-tests or chi-square tests, as appropriate.

To assess label acceptability, the percentage of support for each label was calculated for all variables related to liking, attractiveness and perceived cognitive workload. To compare label performance with respect to the participants’ understanding of nutritional quality, the percentage of correct answers was calculated and chi-square tests were performed. Differences across product categories and across subgroups (e.g. sex, age) were also assessed by using chi-square tests.

The data were weighted according to the French population socio-demographic distribution. Weighting was calculated separately for each sex using an iterative proportional fitting procedure and the 2009 national Census data on age, educational level, area of residence and whether or not the household included any children [32].

All tests of significance were two-sided, and a P value <0.05 was considered significant. Statistical analyses were performed using SAS software (version 9.3; SAS Institute Inc.).

## Results

### Characteristics of the sample

A total of 15,002 participants completed the questionnaire. A total of 772 participants were excluded because in more than two thirds of the cases they had responded “I don’t know”; and 652 participants were excluded because of missing data required for the weighted analyses. This left 13,578 participants available for analyses. Characteristics of included participants are presented in **Table 1**. Compared with excluded participants, included participants were more often women (p<0.0001), younger (p<0.0001), and had a higher educational level (p<0.0001).

**Table 1:** Socio-demographic characteristics of the sample (n = 13,578) (The NutriNet-Santé study, 2014).

	%
**Sex**	
Women	52.4
Men	47.6
**Age**	
18–30	18.9
30–50	33.7
50–65	28.9
>65	18.5
**Occupational category**	
Blue-collar, manual workers	31.2
Intermediate profession/office staff	14.5
Self-employed, farmer	4.5
Managerial staff	9.1
Without professional activity	8.8
Student	4.5
Retired	27.4
**Educational level**	
Up to secondary	75.1
Some college	11.9
University degree	13.0

### Acceptability of front-of-pack labels

**Fig 2:**
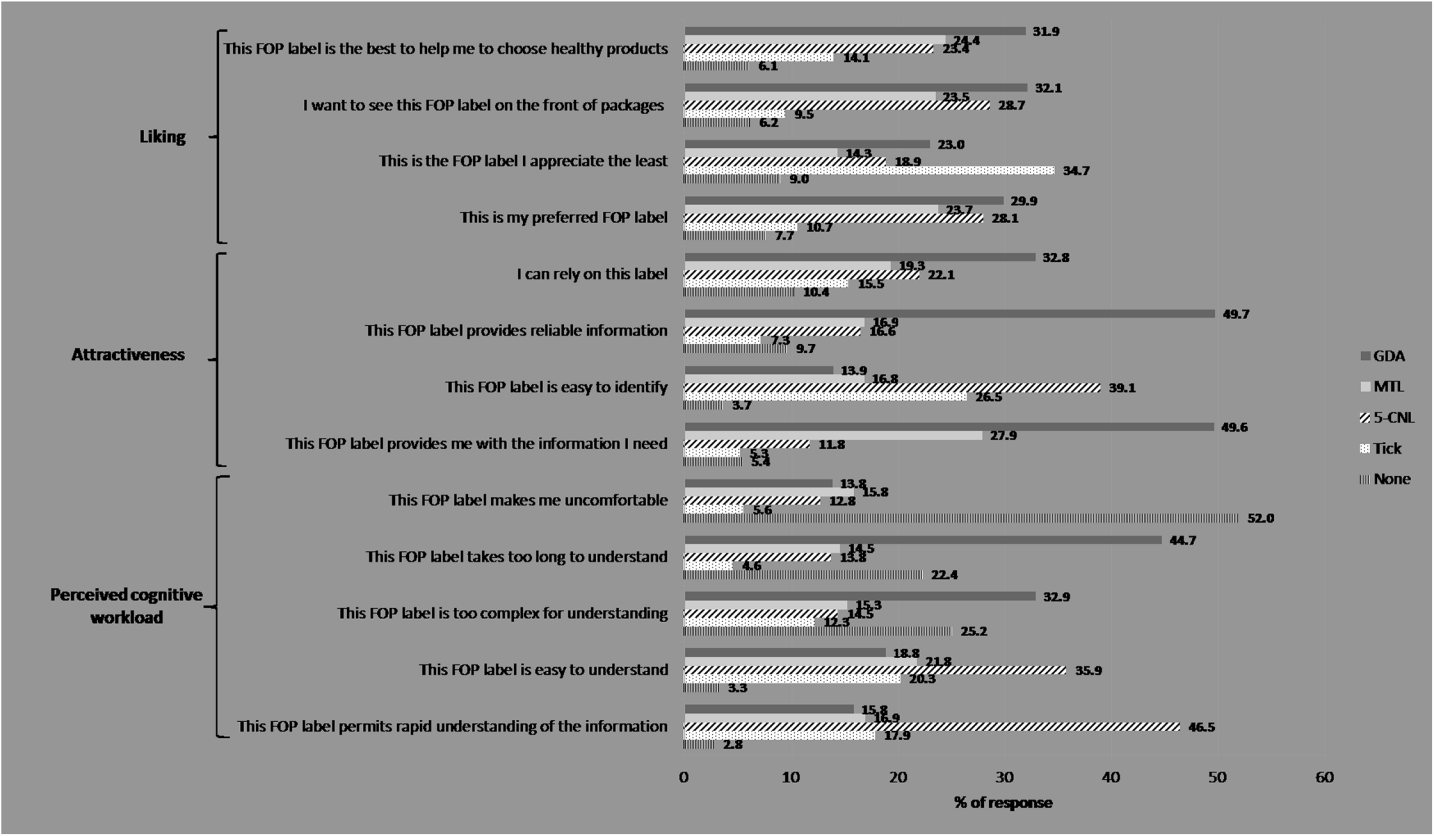
Acceptability of the different labels (n = 13,578) (The NutriNet-Santé study, 2014).

The results on the acceptability of the different FOP labels are presented in **Fig 2**.

#### Liking

GDA was declared to be the preferred FOP label, allowing the choice of healthy products (31.9%), followed by MTL (24.4%), 5-CNL (23.4%) and Tick systems (14.1%). The GDA was also the label that participants wanted to see the most on front of packages (32.1%), followed by 5-CNL (28.7%), MTL (23.5%) and Tick labels (9.5%). The same ranking was observed for the preference of the labels. In turn, when asking about the least appreciated label, the Tick label came first, followed by GDA, 5-CNL and MTL.

#### Attractiveness

Although the GDA label was considered as the label which most contributed to information (49.6% of the respondents) and which provided the most reliable information (49.7%), it was also declared as the least easy to identify (13.9%). The 5-CNL label was the FOP that was the easiest to identify (39.1%).

#### Perceived cognitive workload

The heaviest cognitive workload, in terms of complexity and processing time, was observed for the GDA (32.9% and 44.7%, respectively). On the contrary, the 5-CNL label was the most likely to be found easy and quick to understand (35.9% and 46.5% of the respondents, respectively). Over half of the participants reported that none of the presented labels made them uncomfortable. The highest proportion of discomfort was observed for the MTL (15.8%) and the lowest for the Tick label (5.6%).

### Objective understanding of front-of-pack labels

Results regarding the objective understanding, tests for differences across product categories, and socio-demographic sub-groups are presented in **Table 2**.

**Table 2:** Percentage of correct answers by label, across product categories and socio-demographic characteristics (n = 13,578) (The NutriNet-Santé study, 2014).

	No label	GDA	MTL	5-CNL	Tick	p-value*
	(%)	(%)	(%)	(%)	(%)	
Total	14.6^e^	50.2^c^	56.4^b^	64.6^a^	29.4^d^	<0.0001
**Category of products**						
Prepared fish dishes	23.2^d^	58.3^b^	**69.6** ^**a**^	**67.2** ^**ab**^	41.5^c^	**<0.0001**
Pizzas	4.0^d^	47.8^b^	43.7^b^	**69.1** ^**a**^	29.4^c^	**<0.0001**
Dairy products	20.2^d^	**64.0** ^**ab**^	55.9^b^	**71.5** ^**a**^	40.7^c^	**<0.0001**
Breakfast cereals	14.4^d^	58.1^bc^	60.0^ac^	**60.5** ^**ab**^	23.5^d^	**<0.0001**
Appetizers	11.7^c^	24.2^b^	**50.8** ^**a**^	**54.7** ^**a**^	12.1^c^	**<0.0001**
**Sex**						
Women	16.2^d^	55.2^b^	53.9^b^	**62.9** ^**a**^	32.1^c^	**<0.0001**
Men	12.8^d^	44.6^b^	**59.1** ^**a**^	**66.6** ^**a**^	26.5^c^	**<0.0001**
**Age**						
18–30	14.6^e^	62.5^c^	71.8^b^	**77.4** ^**a**^	39.7^d^	**<0.0001**
30–50	17.7^e^	55.9^c^	59.7^b^	**72.6** ^**a**^	30.3^d^	**<0.0001**
50–65	13.3^e^	41.0^c^	54.6^b^	**59.8** ^**a**^	24.2^d^	**<0.0001**
>65	11.0^d^	41.7^b^	37.3^b^	**44.4** ^**a**^	25.6^c^	**<0.0001**
**Occupational category**						
Blue-collar, manual workers	11.7^d^	46.3^b^	56.4^b^	**68.7** ^**a**^	29.9^c^	**<0.0001**
Intermediate profession/office staff	14.8^e^	57.6^bc^	**69.4** ^**ab**^	**74.0** ^**ac**^	31.8^d^	**<0.0001**
Self-employed, farmer	36.3	56.9	57.0	60.4	38.7	0.48
Managerial staff	17.6^d^	61.6^b^	68.1^b^	**75.8** ^**a**^	30.4^c^	**<0.0001**
Without professional activity	18.8^e^	53.3^bc^	53.2^ac^	**65.0** ^**ab**^	33.0^d^	**<0.0001**
Students	13.6^e^	72.1^bc^	**75.2** ^**ab**^	**77.7** ^**ac**^	33.0^d^	**<0.0001**
Retired	12.0^d^	41.2^b^	43.5^ab^	**49.7** ^**a**^	24.1^c^	**<0.0001**
**Educational level**						
Up to secondary	13.3^d^	46.7^b^	52.1^b^	**61.3** ^**a**^	28.5^c^	**<0.0001**
Some college	19.9^d^	60.0^b^	**70.8** ^**a**^	**74.1** ^**a**^	34.2^c^	**<0.0001**
University degree	17.2^e^	61.2^c^	67.7^b^	**75.3** ^**a**^	30.5^d^	**<0.0001**

*p-values are based on chi-square test

Percentages of correct answers with the same letter were not significantly different.

Boldface indicates the highest percentage of correct answers.

#### Overall understanding

All labels significantly increased the percentage of correct answers compared to the “no label” situation. Overall, when taking into account all product categories, the 5-CNL was the most effective label in terms of permitting a correct ranking of the three products according to their overall nutritional quality (64.6% of correct answers). The MTL label was the second most effective system (56.4%), followed by the GDA (50.2%) and the Tick (29.4%) labels.

#### Understanding across product categories

Irrespective of the product category, the highest percentages of correct responses were observed with the graded label (i.e. 5-CNL) and the nutrient-specific labels (i.e. MTL or GDA) whereas the Tick label exhibited significantly lower percentages of correct answers. For pizzas, the 5-CNL performed significantly better than the other labels in terms of correctly ranking the products in terms of their nutritional quality. Within the prepared fish dishes and the appetizer categories, the MTL and 5-CNL performed significantly better than did the other labels, while for the dairy products category, the 5-CNL and GDA labels performed best. Finally, within breakfast cereals, 5-CNL, MTL and GDA were the most effective labels.

#### Understanding across socio-demographic subgroups

For men, the highest percentage of correct answers was observed with the 5-CNL and MTL labels, followed by the GDA and Tick labels. For women, the 5-CNL system performed better than did the other labels when ranking the products. GDA and MTL were not significantly different with respect to their effect on product ranking and produced the second highest level of correct answers, followed by Tick. On average, women had a higher level of correct answers compared to men.

For all age groups, the 5-CNL label was the most effective label to rank the products according to their overall nutritional quality. It was followed by MTL, GDA and Tick labels, except for participants aged 65 and over, for whom MTL and GDA performed similarly. Overall, younger participants had a higher level of correct responses compared to their older counterparts. For all educational levels, the 5-CNL label produced the highest level of correct responses, followed by MTL, GDA and Tick labels. For individuals with a low education level, no significant difference was found between GDA and MTL. Regarding individuals with an intermediate educational level, no significant difference between the number of correct answers produced for 5-CNL and MTL was detected. On average, participants with a higher educational level (i.e. ‘some college’ and ‘university degree’) had a higher percentage of correct answers.

For all occupational categories, the 5-CNL label produced the highest percentage of correct answers, followed by MTL and GDA. However, differences between 5-CNL and MTL were not significant in five out of seven cases and differences between GDA and MTL were never significant.

## Discussion

The present study provides new insights in the field of consumer acceptability and understanding of different FOP labeling systems. Our study included the three label formats currently in use (i.e. nutrient-specific, graded and simple summary systems), which have been relatively little assessed all together in previous studies. In terms of label acceptability, no single system clearly emerged as the best solution. GDA was declared to be liked and was found to be attractive, yet not easy to identify and to understand. The 5-CNL was considered as the easiest to identify and to understand.

As regards objective understanding, our results indicate that FOP labels increase consumers’ ability to identify healthier food products compared to a “no label” situation. Among the FOP labels tested, the 5-CNL label performed best to compare the nutritional quality of different foods, followed by MTL, GDA and Tick labels. Similar findings were observed across socio-demographic characteristics.

### Acceptability of front-of-pack labels

The GDA was declared to be the preferred label. Previous studies have reported mixed results on consumers’ perception of GDA. In line with our findings, one study conducted in Canada showed that consumers preferred GDA compared to other labels including MTL and simple summary labels [33]. By contrast, other previous studies conducted among shoppers from New-Zealand and Europe found that GDA was the least preferred label [3,13]. A potential explanation for this discrepancy is that the GDA label had been in use on the Canadian and French markets for a long time when studies were performed. The GDA might therefore be preferred because it is familiar. In our study, the GDA label was the one contributing the most to needed information and also the most reliable according to the subjects, which could also explain why it was liked. Despite these strengths, the GDA label presented some weaknesses already highlighted in the literature: it was perceived by the participants as the one requiring the highest cognitive workload in terms of complexity and needed processing time [3,5,15]. In addition, subjects indicated that it was the least easy to identify.

By contrast, the 5-CNL was found to be the easiest label to identify, requiring the lowest cognitive workload in terms of time and complexity. Consistent with these results, other studies have shown that consumers’ processing time was faster when they were exposed to summary labels compared with nutrient-specific labels [3,5,14,15]. In particular, one study including a graded labeling system found that such a label allowed the consumer to identify the healthier product faster than did the other labels [3]. Another explanation to account for the higher performance of the 5-CNL might be its color-coded format and the associated letters which have been shown to focus attention [34] and to help consumers process FOP labels more quickly [5,35]. Focusing the consumer’s attention is of major importance because it is the first step when leading individuals to use nutritional information and making healthier food purchases [4]. Moreover, the label’s ease of identification is all the more important given that in a real shopping situation, consumers are making decisions quickly: European consumers spend, on average, between 25 and 47 seconds when choosing a product in a supermarket [36].

In contrast with previous studies showing that MTL was appreciated by consumers because it was rapid and easy to identify, understand and use, compared with both complex formats (such as GDA) and simple summary labels (such as Tick) [4,12,17], in our study, MTL showed no greater performance compared to other labels. One hypothesis to explain this result is that few studies have compared MTL with graded label formats (such as 5-CNL). Among the tested labels, the MTL and 5-CNL entailed both positive and negative opinions. When comparing these labels, a potential explanation for the fact that the 5-CNL label performed better than did the MTL could be that consumers appreciated simplification [37,38].

However, in their review, Grunert and Wills highlighted that even if consumers appreciate simplification, they would like to know what the simplified information reflects [4], which could explain why Tick was the least preferred label.

Besides, Tick was the least likely to elicit discomfort compared with other labels. Nonetheless, the overall discomfort provoked by the labels was low for all labels. Thus, according to our study, labels providing both positive and negative evaluation of foods (i.e. MTL or 5-CNL) do not cause any more discomfort to the consumer compared with those providing a neutral evaluation, such as the GDA.

### Objective understanding of front-of-pack labels

In line with previous research, we found that FOP labels were an effective tool to help consumers identify food products with the best nutritional quality compared with a no-label condition [5,11,12,16,20,21] and that a graded label format, the 5-CNL, performed better than did other FOP labels [3]. As discussed above, a potential explanation for the better performance of the 5-CNL label might be that these formats include color and text, which have been shown to enhance consumers’ ability to compare the nutritional quality across different products [5,12,38].

On the whole, we observed the second higher percentage of correct answers for the MTL labeling system, followed by GDA and Tick labels. Consistent with our results, a recent review has shown that color-coded labels such as MTL performed better than did GDA to compare products in terms of their healthfulness [5].

In our study, the Tick label demonstrated low performance whereas in other contexts, simple summary labels have been identified as effective systems allowing the comparison across products in terms of their overall nutritional quality [3,13,14,39]. This discrepancy might be explained by the fact that most of the studies in the literature asked participants to compare only two products. In these cases, a summary label format indicating the healthiest product might easily allow subjects to compare products in terms of their healthfulness if only one of the two products has the label. In turn, the design of our study, including a set of three products, highlighted this limit inherent in simple summary labeling systems.

In line with a previous study which included three graded systems (Stars, Smileys and Health Protector Factor), along with MTL, GDA and a simple summary format, we found that the graded label (i.e. 5-CNL) was the label format which exhibited the best effectiveness across product categories [3].

The evaluation of label understanding across population subgroups (sex, age, educational level, occupational category) showed that the 5-CNL label was a particularly effective label allowing the comparison of products according to their nutritional quality. Among subjects with lower educational levels, who have been identified as having potentially more difficulties in understanding nutrition labels [2], the 5-CNL label was the one which demonstrated the greatest performance in terms of increasing the number of correct answers compared with other labels. These results are in line with the literature: in comparison with nutrient-specific labeling systems, simpler FOP labels have been shown to be more effective to help individuals with low formal education to identify healthier products [5,6,14,18].

### Strengths and limitations

This study provides new insights on graded summary labels which have been rarely studied in comparison to nutrient-specific and simple summary formats, whereas the Institute of Medicine, after reviewing the literature, recommended the use of such graded summary labels [14]. Another strength of this study was its large sample size including subjects with various socio-demographic profiles. In addition, we used a weighting scheme to be able to apply these results to the general French population. To avoid any potential effects of the order in which the labels was presented, we set up a rotation system. Possible interactions with the product categories were also taken into account by using all label/product category combinations. Next, we used an objective measure of participant understanding and we developed an experiment including three different products, which is more similar to a real shopping situation than a choice between two products, and also limits the impact of random responses. This design allowed us to identify the limitations of a simple summary label, which provides information on the healthiest product but which failed to differentiate other products. Some limitations in the present study should be noted. First, because the subjects were volunteers in a nutritional study, they may have greater nutrition knowledge and be more interested in nutritional issues. Thus, even though our analyses were weighted on socio-demographic variables, caution is needed when generalizing our results. Secondly, self-reported data may not fully reflect real shopping situations where environmental parameters such as noise, time pressure, surge of commercial messages, prices and special offers may condition the perception, use and understanding of the labels [8,40,41]. Next, compared to other studies, we found a lower number of correct answers regarding nutritional quality. Two hypotheses might be formulated to explain this finding. First, as previously discussed, our test included three products whereas most of the studies asked the participants to compare only two products, which is an easier task. Further, it is possible that some individuals misunderstood the statement « from your point of view » and did not use the labels to order the products according to their nutritional quality leading to a lower rate of correct answers. In addition, when products are correctly ranked, this would be due to knowledge of product or nutrition rather than due to the label, and therefore do not reflect true understanding. Finally, label familiarity was not assessed although this is likely to influence participants’ understanding. However, the GDA label which is the only label in use on the French market and is estimated to be provided on about 20% of the front of food packages in 2010 [42] did not perform better than other, suggesting a limited effect of familiarity.

## Conclusion

Our study supports the fact that nutritional FOP labeling systems could be effective instruments to guide consumers in their food choices. No system was identified as the most appropriate for all studied dimensions of acceptability. Although GDA was ranked best for attractiveness and liking, it presented major weaknesses potentially limiting its effectiveness in a real shopping situation: it was not easy to identify and required a high cognitive workload. By contrast, the 5-CNL label was the easiest label to identify and to understand. In addition, this label was the most effective when testing consumer objective understanding irrespective of the socio-demographic characteristics of the subjects. Our findings provide new insights about acceptability and understanding of labeling systems and supported graded labels such as 5-CNL as particularly effective to guide consumers in their food choices.

## Supporting Information

S1 Appendix Nutrient values and label categorizations of the food products included in the study. (DOCX)

S1 Fig Score computation for 5-CNL attribution. Footnotes: Exceptions were made for cheese, fat, and drinks, in order to better rank items from these food groups according to their nutrient profile, consistently with nutritional recommendations. The percentage of fruits and vegetables was calculated by taking into account fruits, legumes and vegetables as defined in the PNNS (the French nutritional and health policy). Tubers, oleaginous fruits, dried fruits and olives are therefore not considered in this computation. The FSA score allocates different thresholds for fibers, depending on the measurement method used. NSP cut-offs were used to compute fibers score.
(TIF)

## Abbreviations

FOP: Front-Of-Pack
GDA: Guideline Daily Amounts
MTL: Multiple Traffic Lights
UK: United Kingdom
Tick: Green Tick label
5-CNL: Five Color Nutrition Label
FSA: Food Standards Agency
