# Supplementary material for: Effectiveness of Front-Of-Pack Nutrition Labels in French Adults: Results from the NutriNet-Santé Cohort Study
Source: PLoS One. 2015 Oct 28;10(10):e0140898. doi: 10.1371/journal.pone.0140898 (PMC4624978; doi:10.1371/journal.pone.0140898)
Supplement: S1 Appendix — (DOCX) [file pone.0140898.s001.docx]

|  |  | **Nutrition facts (for 100 g of product)** | | | | | | | | | **GDA (% per serving)** | | | | | **MTL** | | | | **5-CNL** | **Tick** |
| --- | --- | --- | --- | --- | --- | --- | --- | --- | --- | --- | --- | --- | --- | --- | --- | --- | --- | --- | --- | --- | --- |
| **Food product** | **Brand** | **Kcal** | **KJ** | **Sugars** | **Lipids** | **SFA** | **Sodium** | **Proteins** | **Fibers** | **Fruits, Veg and Nuts** | **Calories** | **Sugars** | **Fat** | **SFA** | **Sodium** | **Sugars** | **Fat** | **SFA** | **Salt** | **color** |  |
| **PREPARED FISH DISHES** |  |  |  |  |  |  |  |  |  |  |  |  |  |  |  |  |  |  |  |  |  |
| Papillote de cabillaud, duo de carottes et fenouil, jus d'agrumes | Picard | 77 | 320 | 2,3 | 4,5 | 0,7 | 220 | 6,1 | 1,5 | 61 | 12 | 8 | 19 | 11 | 28 | Low | Medium | Low | Medium | Green | yes |
| Colin d'Alaska sauce au beurre | Picard | 116 | 485 | 1,5 | 4,7 | 3,1 | 264 | 6,4 | 0,9 | 10,9 | 23 | 7 | 27 | 62 | 44 | Low | Medium | Medium | Medium | Yellow | yes |
| Saumon et écrasée de pommes de terre à l'huile d'olive (2%) | Picard | 168 | 700 | 1,5 | 12 | 5,2 | 230 | 7,5 | 1 | 10 | 29 | 6 | 60 | 91 | 34 | Low | Medium | High | Medium | Orange | no |
| **PIZZAS** |  |  |  |  |  |  |  |  |  |  |  |  |  |  |  |  |  |  |  |  |  |
| Lardons, pizza au fromage de chèvre | Carrefour | 239 | 1002 | 3,4 | 8,5 | 3,6 | 433 | 11 | 2,4 | 0 | 20 | 6 | 21 | 31 | 31 | Low | Medium | Medium | Medium | Yellow | yes |
| 4 fromages | Carrefour | 268 | 1122 | 3,9 | 12 | 8,4 | 470 | 13 | 2,3 | 0 | 23 | 7 | 29 | 71 | 34 | Low | Medium | High | Medium | Fushia | no |
| Regina | Carrefour | 246,8 | 1031 | 8 | 12,4 | 4 | 460 | 10,5 | 2,6 | 41 | 21 | 15 | 30 | 34 | 33 | Medium | Medium | Medium | Medium | Orange | no |
| **DAIRY PRODUCTS** |  |  |  |  |  |  |  |  |  |  |  |  |  |  |  |  |  |  |  |  |  |
| Fromage blanc | Intermarché | 72 | 305 | 3,8 | 3,2 | 1,9 | 80 | 7,3 | 0 | 0 | 4 | 4 | 5 | 10 | 3 | Low | Medium | Medium | Low | Green | yes |
| Yaourt brassé | Intermarché | 68 | 284 | 5,3 | 3,2 | 2,1 | 39 | 4 | 0 | 0 | 4 | 7 | 6 | 13 | 2 | Medium | Medium | Medium | Low | Yellow | yes |
| Yaourt à la grecque | Intermarché | 116 | 485 | 4,7 | 9,2 | 6,4 | 24 | 3,7 | 0 | 0 | 9 | 8 | 20 | 48 | 2 | Low | Medium | High | Low | Orange | no |
| **APPETIZERS** |  |  |  |  |  |  |  |  |  |  |  |  |  |  |  |  |  |  |  |  |  |
| Cacahuètes, sans sel ajouté | Auchan | 643 | 2687 | 2,9 | 53,5 | 7,4 | 0 | 26,3 | 9,5 | 100 | 6 | 1 | 15 | 7 | 0 | Low | High | High | Low | Yellow | yes |
| Chips saveur bolognaise | Auchan | 552 | 2307 | 2 | 34 | 3,1 | 787 | 6,4 | 5 | 0 | 7 | 1 | 12 | 4 | 8 | Low | High | Medium | High | Fushia | no |
| Mini-flûtes feuilletées emmental | Auchan | 497 | 2077 | 1,4 | 23 | 15 | 827 | 13 | 2,8 | 0 | 6 | 0 | 8 | 19 | 9 | Low | High | High | High | Red | no |
| **BREAKFAST CEREALS** |  |  |  |  |  |  |  |  |  |  |  |  |  |  |  |  |  |  |  |  |  |
| Muesli noisette noix de coco bio | Leclerc | 370 | 1570 | 18 | 8,5 | 3 | 36 | 10 | 10 | 0 | 6 | 6 | 4 | 5 | 0 | High | Medium | Medium | Low | Yellow | yes |
| Muesli croustillant bio chocolat | Leclerc | 440 | 1850 | 24 | 14 | 8,5 | 25 | 9,5 | 8,5 | 0 | 7 | 8 | 6 | 13 | 0 | High | Medium | High | Low | Fushia | no |
| Muesli croustillant | Leclerc | 460 | 1920 | 23 | 19 | 210 | 1 | 7 | 0 |  | 7 | 8 | 8 | 18 | 3 | High | Medium | High | Medium | Red | no |

SFA : Saturated fatty Acids ; GDA: Guideline Daily Amounts; MTL: Multiple Traffic Lights; Tick: Green Tick label; 5-CNL: Five Color Nutrition Label
