# Supplementary figures and images for: Effectiveness of Front-Of-Pack Nutrition Labels in French Adults: Results from the NutriNet-Santé Cohort Study
Source: PLoS One. 2015 Oct 28;10(10):e0140898. doi: 10.1371/journal.pone.0140898 (PMC4624978; doi:10.1371/journal.pone.0140898)

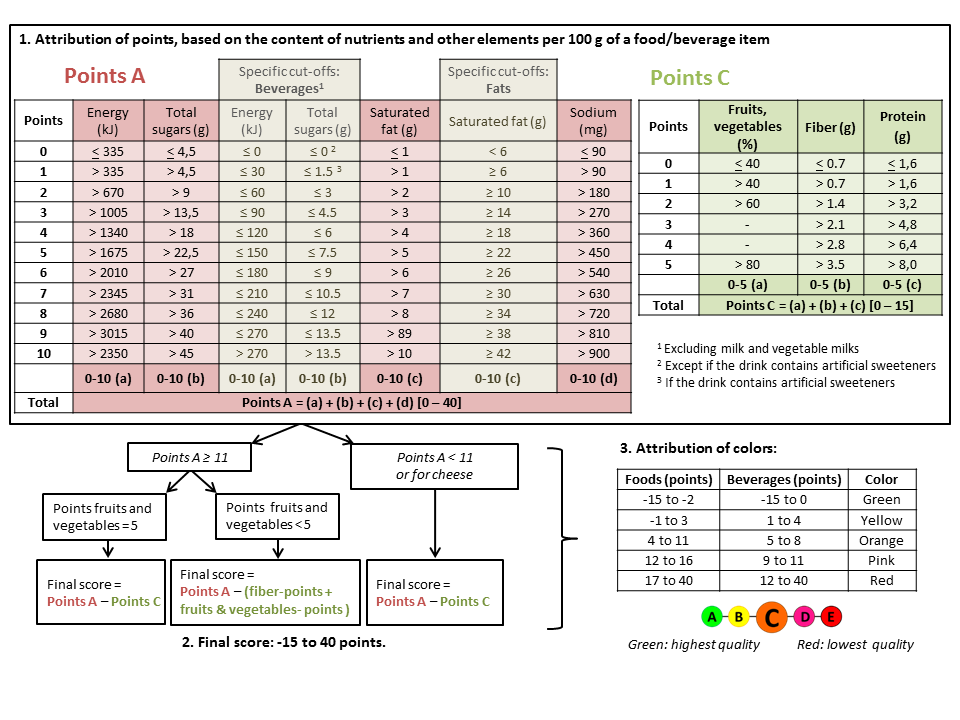

Supplement: S1 Fig — Footnotes: Exceptions were made for cheese, fat, and drinks, in order to better rank items from these food groups according to their nutrient profile, consistently with nutritional recommendations. The percentage of fruits and vegetables was calculated by taking into account fruits, legumes and vegetables as defined in the PNNS (the French nutritional and health policy). Tubers, oleaginous fruits, dried fruits and olives are therefore not considered in this computation. The FSA score allocates different thresholds for fibers, depending on the measurement method used. NSP cut-offs were used to compute fibers score. (TIF) [file pone.0140898.s002.tif]
